# Mucosal Injury during Anti-Cancer Treatment: From Pathobiology to Bedside

**DOI:** 10.3390/cancers11060857

**Published:** 2019-06-20

**Authors:** Debora Basile, Paola Di Nardo, Carla Corvaja, Silvio Ken Garattini, Giacomo Pelizzari, Camilla Lisanti, Lucia Bortot, Lucia Da Ros, Michele Bartoletti, Matteo Borghi, Lorenzo Gerratana, Davide Lombardi, Fabio Puglisi

**Affiliations:** 1 Department of Medicine (DAME), University of Udine, 33100 Udine, Italy; deborabasile1090@gmail.com (D.B.); carla.corvaja@cro.it (C.C.); silvioken.garattini@cro.it (S.K.G.); giacomo.pelizzari@cro.it (G.P.); camilla.lisanti@cro.it (C.L.); lucia.bortot@cro.it (L.B.); michele.bartoletti@cro.it (M.B.); fabio.puglisi@cro.it (F.P.); 2 Department of Medical Oncology, Centro di Riferimento Oncologico di Aviano (CRO), IRCCS, 33081 Aviano, Italy; paola.dinardo@cro.it (P.D.N.); lucia.daros@cro.it (L.D.R.); davide.lombardi@cro.it (D.L.); 3 Intensive Care Unit, Centro di Riferimento Oncologico di Aviano (CRO), IRCCS, 33081 Aviano, Italy; matteo.borghi@cro.it

**Keywords:** mucosal injury, mucositis, mucosal impairment, anti-cancer treatments

## Abstract

Mucositis is one of the most common debilitating side effects related to chemotherapy (CT), radiation therapy (RT), targeted agents and immunotherapy. It is a complex process potentially involving any portion of the gastrointestinal tract and injuring the mucosa, leading to inflammatory or ulcerative lesions. Mechanisms and clinical presentation can differ according both to the anatomic site involved (oral or gastrointestinal) and the treatment received. Understanding the pathophysiology and management of mucosal injury as a secondary effect of anti-cancer treatment is an important area of clinical research. Prophylaxis, early diagnosis, and adequate management of complications are essential to increase therapeutic success and, thus, improve the survival outcomes of cancer patients. This review focuses on the pathobiology and management guidelines for mucositis, a secondary effect of old and new anti-cancer treatments, highlighting recent advances in prevention and discussing future research options.

## 1. Introduction

Mucositis is one of the most common debilitating side effects related to chemotherapy (CT), radiation therapy (RT), targeted agents and immunotherapy [1,2]; it occurs in 20–40% of the patients receiving anti-cancer treatments for solid tumors, 60–80% of the ones undergoing hematopoietic stem cell transplantation (HSCT), and is experienced by almost all patients receiving RT for head and neck cancers (HNC) [3,4,5]. Mucositis is a complex process that leads to inflammatory and/or ulcerative lesions. It can potentially involve any portion of gastrointestinal tract and injures mucosa through the interplay of the epithelial, mesenchymal and immune cells. The specific mechanism of this process, along with the clinical presentation, can differ according both to the anatomic site involved (oral or gastrointestinal) and the causal anti-cancer therapy [6,7,8].

Oral mucositis (OM) is characterized by erythema, ulceration, pain and, eventually, bleeding; the loss of integrity of the oral mucosa can favor local and systemic infections that can compromise nutrition and fluid intake [3,9,10]. On the other hand, gastrointestinal mucositis (GIM) is sometimes responsible for nausea, vomiting and diarrhea. The onset, timing and clinical presentation are also influenced by patient-related risk factors. Among these, age, ethnicity, gender, and disorders such as malnutrition and poor oral health can increase the risk of developing mucositis [6,7,8,11,12]. Moreover, patients with underlying systemic illnesses, such as autoimmune diseases or diabetes, are more likely to have an altered tissue environment, which can further predispose them to mucosal damage [3].

Many studies have shown that the risk of mucositis increases along with the intensity of the therapy. Furthermore, combination treatment (e.g., RT with concurrent CT used for HNC) may boost the onset of mucositis [13]. An adequate classification of lesions is crucial to the ability to choose the most suitable management and, eventually, appropriate dose modifications of cancer therapy. Understanding the pathophysiology and management of mucosal injury as a secondary effect of anti-cancer treatment is essential to increase therapeutic success and, thus, improve the survival outcomes of cancer patients.

Mucositis may have a severe impact on a patient’s quality of life (QoL) and negatively influences the ability to maintain the proper schedule and intensity of anti-cancer treatment. Moreover, it has a non-negligible economic effect due to the cost of care [14].

This review focuses on the pathobiology and management guidelines for mucositis as a secondary effect of anti-cancer treatment, highlighting recent advances in prevention and discussing future research options. Studies evaluating the pathogenesis of “classical mucositis” induced by CT and RT, pathogenesis of new anti-cancer treatment, risk factors associated with mucositis, prophylaxis, clinical presentation and management of classical and new anti-cancer treatments were included.

## 2. What Lies Beneath?

**Figure 1:**
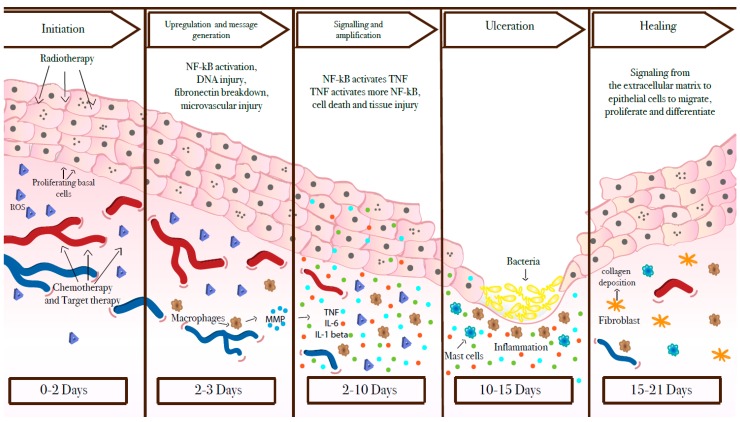
Mucositis pathobiology. This model has proven relevant for oral mucositis and gastrointestinal mucositis induced by chemotherapy and radiotherapy [7,13]. Conversely, there is still little information about the pathogenesis of mucositis associated with the newer anticancer treatment, but they likely differ from “classical mucositis”.

Five stages of pathogenesis have been identified for mucositis: initiation, upregulation/activation, signal amplification, ulceration, and healing (Figure 1) [7,13].

Initiation: This phase is triggered by exposure to anti-cancer procedures, which induces direct damage to the DNA, oxidative stress and, consequently, generates reactive oxygen species (ROS). The injury to epithelial, submucosal and endothelial cells provokes the release of endogenous damage-associated pattern molecules (CRAMPs) [15].

Upregulation/activation: ROS, innate immune response and binding of CRAMPs to receptors propagate further the damage of the cell membranes and activate several transcriptional pathways [15,16]; among these, one of the best known is the pathway of nuclear factor NF-κB [17,18,19]. This latter, once activated, induces an increase in production of pro-inflammatory cytokines, such as tumor necrosis factor-α (TNF-α), interleukin (IL)-6, stress responders such as cyclooxygenase-2 (COX-2), and cytokine modulators by fibroblasts and endothelial cells, which, in turn, can lead to cell apoptosis [20,21,22,23]. In this phase, there is also an increased expression of genes related to adhesion molecules and angiogenesis [18,19].

Amplification: The primary response initiated during the first two phases triggers the expression of several molecules that, in turn, influence local response. NF-κB can be up-regulated by TNF-α, which can initiate mitogen-activated protein kinase (MAPK) signaling, leading to activation of Jun N-terminal kinase signaling. Apoptotic pathways are activated on submucosal and basal epithelial cells causing mucosal ulceration and atrophic transformations. Metalloproteinases (MMPs) too can be dysregulated leading to important pathogenic effects [22,24].

Ulceration: In this phase, clinically symptomatic deep ulcerations appear. In OM, secondary to CT administered by a bolus schedule, the time lapse between initial injury and clinical damage is about 4 days; shortly thereafter, bacterial colonization from both gram-negative and gram-positive bacteria happens; at the same time, CT-induced leukopenia favors progression of the infection. Differently, GIM becomes clinically evident much sooner, within 1–2 days after CT administration.

Healing: In the last stage, there is spontaneous healing of the ulcers, but an increased risk of recurrence due to residual angiogenesis may persist. This final stage is characterized by epithelial proliferation, migration, and differentiation stimulated by the extracellular matrix, and the simultaneous restoration of the local microbial flora [15,25].

## 3. Risk Factors

Like other adverse (treatment-related) events (AEs), the onset of mucositis is influenced by the features of the anti-cancer regimen, as well as by the patient’s characteristics. Patient-related risk factors include genetic polymorphisms, systemic comorbidities, and other general host-related risk factors. The impact of gender on the risk of GIM is well established for patients exposed to 5-fluorouracil (5-FU)-based CT, the risk for women is higher than for men [26,27,28]. Patients in extreme age groups appear to be at higher risk of developing OM, as demonstrated by increased incidence in pediatric and older patients [11,29]. In addition, the elderly seem to be at higher risk for suffering more severe mucositis [30]. Body composition can play a role in drug metabolism and patients with a low, lean body mass, women and other patients with a low BMI, can be exposed to higher levels of drugs and, as a consequence, develop more severe toxicities [31].

### 3.1. Anti-Cancer Treatment

The spectrum of the pathobiology, onset, duration and severity, is particularly wide during anti-cancer treatment, differing according to the adopted regimen, dosage, and delivery schedule. Moreover, risk and grade intensify with the proceeding of treatment for a cumulative effect. More details are provided in the next section.

### 3.2. Genetic Polymorphisms

Polymorphisms in genes codifying for enzymes engaged in drug metabolism have been linked to an increased risk of developing treatment-related toxicities. A higher blood level of 5-FU in patients with dihydropyrimidine dehydrogenase (DPD) deficiency can result in severe or fatal myelosuppression and mucositis [32]. Even patients with polymorphism of the thymidylate synthase (TYMS) gene tend to have more severe toxicity from 5-FU [33]. Patients who carry the 677TT genotype for methylentetrahydrofolate reductase (MTHFR) experience a more severe mucositis when treated with methotrexate for HCT [34]. In addition, polymorphisms in cytokine genes, like TNF, have been associated with a higher severity of CT-related toxicities, including mucositis, in a Japanese population treated with cisplatin and 5-FU [35]. Patients with systemic disease characterized by an increased (e.g., Addison disease) or a reduced apoptosis’s rate (e.g., psoriasis) seem to be, respectively, at higher and lower risk of developing mucositis [36].

More recently, six pathways were identified as being associated with mucositis: PTK6 signaling and the Wnt signaling pathway, two pathways associated with transforming the growth factor (TGF)-b, ERK signaling and the inflammatory response pathway. Of these, the first two pathways seem more specific to mucositis, while the other four are involved in the development of other ailments such as IBS. PTK6 is normally expressed in intestinal cells and is thought to play a role in epithelial barrier function. Suppression of PTK6 is associated with an increase in apoptosis of proliferating cells. On the other side, the Wnt pathways interact with the NK-kB pathway and could be involved in immune response and antigen presentation. Activation of the TGF-b signaling pathway is associated with treatment-related injury, and its regulation through Smad7 or inhibitions of b1 integrins changes the subsequent development of mucositis. The ERK signaling pathway acts in conjunction with extra- and intracellular signaling events, while the inflammatory response pathway is likely linked to the activation of the innate immune response. It is interesting to note how these pathways are shared with diseases with similar clinical presentation (such as IBS), therefore lending strength to the hypothesis that risk for development of a certain clinical phenotype (i.e., diarrhea) is related to a patient specific and individual genomic profile [37].

### 3.3. Role of Microbial Flora

There is no conclusive evidence that oral and intestinal microbial flora play an active role in the development of mucositis. However, it is known that microbiota can stimulate the production of pro-inflammatory cytokines [38]. At the same time, probiotic bacteria can promote the production of cytoprotective pathways [39]. Moreover, changes in the composition of luminal microbiota can result in proliferation of pathogenic species, thus providing a chance for bacteremia and sepsis through the disruption of the epithelium following a decrease in the immune response. However, microbiota play a key role in activating immune cells including natural killer (NK) cells, mast cells, macrophages and dendritic cells by recognizing pathogen-associated molecular patterns (PAMPS). Then, Toll-like receptors (TRL) recognize these molecules, which activate NF-κB and stimulate the production of pro-inflammatory cytokines. These may, in turn, recruit leukocytes and stimulate them to further secrete more pro-inflammatory cytokines [40]. During anti-cancer treatment, microbial composition may become imbalanced and, thus, the physiological microbiota may become underrepresented (dysbiosis) [41]. The entity and type of variations in microbiota composition differ along the gastrointestinal tract, according to the CT regimen, schedule and doses. Notably, these changes are potentially involved in initiating and exacerbating mucosal impairment.

### 3.4. Role of the Innate and Adaptive Response in Mucosal Injury 

Immune response is involved in maintaining pathophysiological homeostasis. However, it could also induce regimen-related toxicities during anti-cancer therapy. Both in OM and GIM, a key role is played by the innate immune system through the recruitment of inflammatory cells and the interplay among epithelium, dendritic cells, and macrophages [42,43]. More in detail, the anticancer treatment could activate cell injury through the generation of damage-associated molecular patterns (DAMPs) or PAMPs identified by pattern recognition receptors (PRRs), thus increasing pro-inflammatory cytokine production [44,45,46]. Furthermore, GIM alterations in epithelial intestinal barriers promote a rearrangement of microbial flora and bacterial translocation to the intestinal lamina propria, which can enhance neutrophil recruitment and tissue impairment [45,46]. However, even antibody-mediated and cell-mediated immunotoxicity triggered by adaptive immune response are responsible for tissue damage. Guabiraba et al. showed that neutrophils were highly expressed in the blood three days after irinotecan treatment in mice. Moreover, even macrophages play an important role in CT-induced mucositis [47]. Recent evidence showed that T regulatory cells played a critical role in controlling irinotecan-related GIM [48]. Moreover, inflammasome influenced secretion of IL-33 at the level of the intestinal epithelial cells of mice treated with irinotecan. Treatment of mice with an anti-IL33 resulted in attenuation of intestinal damage [49].

## 4. Prophylaxis

Several approaches are currently available for OM and/or GIM prophylaxis [7]. The MASCC/ISOO and European Society of Medical Oncology (ESMO) guidelines provide a systematic review of the recommended or suggested preventive interventions during cancer treatments (Table 1) [50,51].

### 4.1. Basic Oral Care and Topic Therapies for OM and mTOR-Induced-Mucositis Prophylaxis

The first preventive measure checked during cancer treatments is the daily oral hygiene routine, evaluated with a baseline dentist evaluation in order to remove potential sources of infection or trauma. The recommended oral care protocol should include a soft toothbrush twice a day, flossing, and alcohol-free mouthwashes after oral hygiene (with bland 0.9% saline, sodium bicarbonate or plain water). Additionally, dietary and nutritional restrictions should reduce potential irritating stimuli, such as hot and spicy food, dehydration, alcohol and smoking [51,54]. Taking into account specific topical therapies, oral rinses with benzydamine HCl, a non-steroidal anti-inflammatory agent, was shown to be effective in preventing OM in patients with HNC receiving exclusive RT [55]. Similarly, dexamethasone-containing mouthwashes (0.5 mg/5 mL oral solution) are specifically recommended for prophylaxis of mTOR (mammalian target of rapamycin)-induced-mucositis in patients with metastatic breast cancer, even if a proper randomized, placebo-controlled trial is still missing [52].

### 4.2. Physical Therapies for OM Prophylaxis

Several trials have demonstrated the efficacy of oral cryotherapy (intraoral ice-chip therapy) for the prevention of 5-FU induced stomatitis [56,57,58]. As shown by a recent Cochrane meta-analysis, including five trials and 444 patients treated with short-term 5-FU bolus, oral cryotherapy reduced the incidence of all grades and, specifically, severe OM (RR 0.61, 95% CI 0.52–0.72 and RR 0.40, 95% CI 0.27–0.61, respectively) [59]. Moreover, the same meta-analysis examined the data of an additional 271 patients receiving high-dose melphalan-based CT before HSCT, which showed a reduction in all grades of mucositis (RR 0.59, 95% CI 0.35–1.01) and a significant reduction in severe mucositis (RR 0.38, 95% CI 0.20–0.72) [59]. Since both drugs have a short duration, a possible mechanism of action relies on cold-induced vasoconstriction, which could reduce mucosal delivery of CT and therefore its potential local toxicity. Another physical technique investigated for OM prevention was low level laser therapy. It was mainly evaluated in two clinical settings: HSCT [60,61,62,63] and RT for patients with HNC [50,51]. Numerous trials provided consistent evidence that a mucosal pre-treatment with a helium-neon laser was likely to reduce mucosal injury and to promote the epithelial healing process [50,51]. However, there was no consensus on the optimal wavelength, possible detrimental biological effects or on its cost–benefit value [62,64].

### 4.3. Systemic Therapies for OM and GIM Prophylaxis

Mucosal injury can be approached from a preventive systemic point of view. Oral zinc supplements have shown activity in preventing OM in patients with oral cancer who are candidates for CT-RT or RT [50,65,66].

Another preventive option is represented by amifostine, an organic phosphorylated aminothiol administered intravenously, able to recruit ROS scavengers, thus protecting the normal epithelium and connective tissue [67]. The FDA indication for amifostine refers to xerostomia prophylaxis in post-operative HNC patients treated with RT, with conflicting data on OM prevention [53,68]. Its preventive use is also recommended for RT-induced proctitis and esophagitis [50]. Unfortunately, significant side-effects (mainly nausea and hypotension) limited its prescription in clinical practice. Additionally, sulfasalazine, a salicylate used in inflammatory bowel disease management, may be indicated as preventive remedy for RT-induced enteropathy (500 mg orally twice a day) in patients receiving pelvic RT [50,51]. On the contrary, similar compounds such as acetylsalicylic acid or mesalazine have shown no role in this setting.

Finally, despite the lack of definitive data on their mechanisms of action [69], probiotic agents containing the *lactobacillus* species may be of value for diarrhea prophylaxis in patients with pelvic malignancy exposed to CT or RT [50,51], and they are currently being evaluated for prevention of CT-RT-induced OM (NCT01707641) and irinotecan-induced diarrhea (NCT02819960).

## 5. Across Old and New Anti-Cancer Treatments

### 5.1. Chemotherapy Induced Mucositis

Approximately 40% of CT treated patients develop mucositis. The incidence is conditioned by the regimen; antimetabolites (5-FU, capecitabine and S-1), anthracyclines, irinotecan and taxanes are drugs leading to a higher rate of mucositis [1]. Among antimetabolites, S-1 and capecitabine carry a lower risk of mucositis than 5-FU [70]. For regimens such as docetaxel, cisplatin and fluorouracil (TPF), and in combination treatments (such as RT-CT for HNC), OM occurs in over 50% of patients. Mucin reduction seems to be one of the mechanisms underlying OM in platinum-based CT.

As for GIM, even though the etiology of the cellular damage induced by different CT drugs differs, all pathways ultimately converge in the shortening of crypt length, dampening and fusion of villi, enterocyte hyperplasia and increased apoptosis (more commonly located in the small bowel). A role of pro-inflammatory cytokines and proteins involved in apoptosis regulation has been suggested by many studies evaluating diverse cytotoxic agents (5-FU, methotrexate an irinotecan) [16,46].

The pathobiological mechanisms of GIM are similar to those that promote the development of OM; such mechanisms include disruption of tight junctions and matrix metalloproteinase-mediated connective tissue impairment [71,72].

One of the chemotherapeutic drugs more extensively studied in this regard is irinotecan. Irinotecan is a topoisomerase inhibitor that seems to induce mucositis by activating caspases and p53, downregulating the PI3K/Akt pathway, and promoting the MAPK and PKC pathways, which in turn induce specific effects, such as the reduction in goblet cell number and mucin hypersecretion, which contribute to amplification of the magnitude of diarrhea [73]. There are two main clinical presentations of GIM during irinotecan treatment. Activation of the parasympathetic system, the subsequent inhibition of acetylcholinesterase and the release of acetylcholine lead to cholinergic syndrome and early-onset diarrhea. Conversely, both changes in intestinal motility and direct damage to the mucosa induced by cytokines and inflammatory-mediated effects contribute to late-onset diarrhea [46]. GIM occurs more frequently with a combination of irinotecan and fluoropyrimidines, mainly with capecitabine [70]. In fact, patients treated with the capecitabine and irinotecn (XELIRI). regimen reported higher gastrointestinal toxicity than with fluorouracil and irinotecan (FOLFIRI). For regimens such as fluorouracil and oxaliplatin (FOLFOX) or FOLFIRI, GIM is reported to be 50% and 89% respectively [74].

Notably, GIM induced by oxaliplatin and carboplatin tends to have a lower grade toxicity compared to cisplatin [75]. Taxane treatment induces mild or moderate mucositis in 29–63% of patients, mainly in those treated with docetaxel.

Clinical presentation: OM appears shortly after the first cycle with gradual recovery 2–3 weeks after the discontinuation of treatment [8]. The clinical course may be protracted when complicated by infection, in particular if associated with severe neutropenia [6]. Nausea, vomiting, dysphagia and dyspepsia, with or without pain, can be caused either by infections such as candidosis or, less commonly, as a direct effect of treatment [76]. GIM is usually acute, with rapid onset of diarrhea (generally within 24–48 h of treatment), abdominal pain, nausea, vomiting, anorexia and, in severe cases, weight loss, dehydration and sepsis [24,77].

Management: Different strategies have been tested and are currently under evaluation for the treatment of CT-induced mucositis. However, few agents have been approved while for most of them the evidence is not sufficient to establish a standard therapy [78].

In standard-dose CT-induced OM, studies have failed to determine a benefit of chlorhexidine. No significant difference in the ratings and duration of pain was observed in a double-blind clinical trial conducted on 23 patients receiving CT and evaluating the effectiveness of a standardized oral care protocol (PRO-SELF) plus mouthwash, salt and soda rinses, and chlorhexidine [79].

The efficacy of sucralfate for established OM needs further evidence. Sucralfate is an aluminum salt that protects mucosa from mechanical damage. It also prevents the release of inflammatory cytokines and stimulates angiogenesis, fibroblast, and epidermal cell proliferation contributing to tissue repair. Its beneficial effect has only been observed as prophylaxis for patients treated with 5-FU [80].

Topical vitamin E could be beneficial in reducing the severity of OM but no therapeutic gain would be achieved by using systemic vitamin E in this setting [81].

The increasing knowledge of mechanisms underlying mucositis allows us to consider the use of antioxidant agents as a potential interventional method.

In regard to GIM, the efficacy of octreotide after loperamide failure in 5-FU-induced diarrhea has been assessed in multiple clinical trials [82,83,84,85,86]. Octreotide is a somatostatin analogue that regulates intestinal water and electrolyte transport, inhibits the release of gastrointestinal hormones and plays a role in the preservation of the epithelial barrier. In patients who develop loperamide-refractory diarrhea after standard or high-dose CT for HSCT, octreotide is recommended at a dose of at least 100 μg, administered subcutaneously twice daily [50].

### 5.2. Radiotherapy-Induced Mucositis

Mucositis is a common side effect in patients treated with RT and CT-RT. Although CT and RT-induced mucositis share the same pathobiological mechanisms, the timing of cellular damage and the onset of symptoms differ; RT induces damage within a few seconds of first exposure and this damage is reiterated in time due to the fractionated schedule. Clinically, symptoms stemming from atrophic changes, such as erythema and soreness, start at the end of the first week of exposure; this is followed, as the cumulative dose increases, by ulceration, which can last for up to 6 weeks after completion of RT [8]. In patients with HNC undergoing concomitant treatment with RT associated with cetuximab, a p16-negative status seems to be associated with a higher rate of grade 3–4 OM [87]. Also the increase of salivary concentration of cytokines (in particular, IL-6 and IL-1β) seems to be related to the severity of mucositis, although there is no relation to baseline concentration [88].

Clinical presentation: Nearly all HNC patients undergoing RT develop some degree of OM. Asymptomatic redness progresses to elevated desquamative patches, which evolve into large, painful contiguous pseudo-membranous lesions associated with dysphagia and, subsequently, a decrease in oral intake [89]. Oral lesions usually appear during the first weeks of treatment and do not disappear until 4–5 weeks after the last administration. The rate and severity of these toxicities increase with concomitant CT-RT [90]. 

Usually, proctitis occurs after RT (either on its own or in combination with CT) in rectal or other pelvic cancers. The onset can be acute (during or within six weeks after RT)—with diarrhea, tenesmus, urgency and mucus discharge—or chronic—with bleeding, symptoms of obstructed defecation and rectal pain [76].

Management: Lacking or conflicting evidence does not allow us to provide guidelines for mouth rinses to treat OM in HNC patients receiving RT [91].

Amifostine has been proven to reduce the incidence of acute and late xerostomia [92,93]. However, no randomized trial has proven its role and a certain toxicity caused by its intravenous administration has limited its clinical use [94].

A deficiency in glutamine levels can negatively affect mucosal resistance. A double-blind, randomized, placebo-controlled trial was conducted in 40 patients receiving CT-RT for HNC, to evaluate the efficacy of oral glutamine in attenuating the seriousness of mucositis. This agent seemed to decrease the incidence of grade 4 mucositis, thus reducing treatment delays, duration of supplemental nutrition and opioid use [95]. However, its clinical use is still not supported by convincing scientific evidence.

In patients undergoing CT-RT for oral cancer, the use of zinc sulphate might be considered as it is crucial to tissue repair processes. A study showed that zinc sulphate, administered daily throughout the duration of CT, reduced xerostomia and pain [66].

Among anti-inflammatory agents, benzydamine HCl oral rinses have demonstrated analgesic, anesthetic and antimicrobial characteristics [96]. Its use has been associated with a decreased severity of ulcerative oral lesions and pain in RT-induced OM, delaying the need for opioids [55].

MASCC/ISOO clinical guidelines for mucositis have recommended against the use of systemic sucralfate in patients with solid tumors receiving RT who develop GIM.

Conversely, sucralfate enemas represent a useful interventional strategy for chronic RT-induced proctitis in patients with rectal bleeding [50]. Interestingly, 37 patients with RT-induced proctitis were randomized to receive a 4-week course of oral sulfasalazine and prednisolone enemas or rectal sucralfate enemas and an oral placebo. Both regimens determined a significant clinical improvement, assessed at endoscopic evaluation [97]. Other therapies for chronic RT proctitis with bleeding include argon beam coagulation, electrocoagulation, formalin treatment, and hyperbaric oxygen treatment [50].

### 5.3. Targeted Therapy-Induced Mucosal Injury

Targeted therapies have reshaped anti-cancer treatment, introducing a new peculiar toxicity profile. Regardless of the specific drug category, patient education on the prophylactic measures and early identification of symptoms and signs are crucial to the management of mucositis.

#### 5.3.1. mTOR Inhibitors

Among targeted drugs, the mTOR inhibitors are capable of causing the worst mucosal damage [98].

mTOR inhibitors avoid the activation of mTOR complex 1 (mTORC1) by binding to FKBP-12 and forming a ternary complex, which regulates cellular metabolism [21,99]. Epithelial injury leads to the release of pro-inflammatory cytokines, such as IL-1, IL-6, IL-8, and the activation of innate and adaptive immune responses. Namely, it causes limited infiltration of T regulatory and cytotoxic cells and upregulation of heat shock protein 27 [99]. Recent studies showed absence of microorganisms in mucosal changes. In phase II and III studies, the overall incidence of everolimus-related stomatitis was about 50%, mostly moderate, although 10% was grade 3/4 [100]. Management of stomatitis depends on the severity of the clinical manifestations. Recent meta-analyses showed that incidence of any grade stomatitis ranges between 33.5–52.9%, and between 4.1–5.4% for grade 3. In the BOLERO-2 trial, treatment with exemestane and everolimus in metastatic breast cancer patients was limited by stomatitis, representing the most common side effect with an all-grade incidence of 67% (33% grade ≥ 2, 8% grade 3), leading to dose reduction or interruption [101,102]. Moreover, it was the second cause of treatment discontinuation [102].

Clinical Presentation: mTOR inhibitor-associated OM (mIAOM) almost entirely concerns the non-keratinized, movable oral surface (lateral tongue, buccal and labial mucosa, soft palate and floor of mouth) with isolated or multiple ulcerations, which are usually smaller (0.5 cm) than the ones induced by CT [21]. Typically, they have a rapid onset (usually within 5 days), mainly during the first cycle of treatment, and can spontaneously improve or resolve despite continuation of mTOR inhibitor treatment [103]. In some patients, it may persist over an extended period of time. Moreover, although usually limited, lesions can be very painful [102].

**Figure 2:**
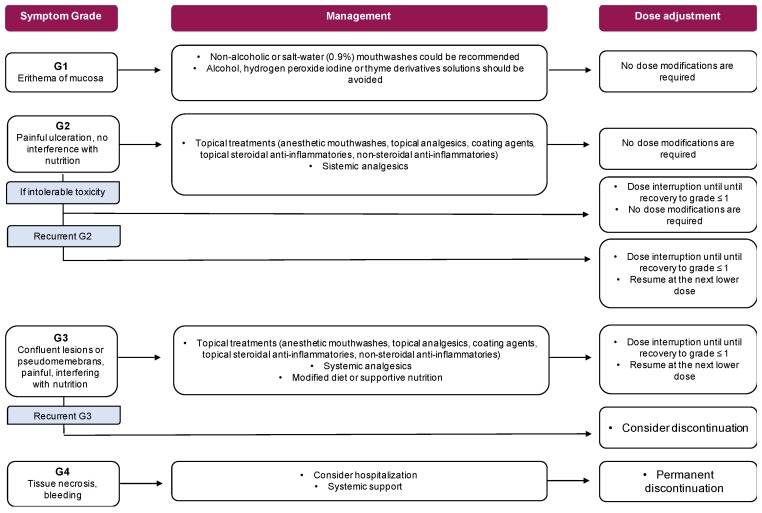
Dose modifications and management of mTOR inhibitors.

Management: Aggressive treatments, such as alcohol, hydrogen peroxide iodine or thyme derivative solutions, should be avoided [102]. The use of non-alcoholic or salt-water (0.9%) mouthwashes is recommended [104]. If high-grade stomatitis occurs, a wide range of treatments could be used, including anesthetic mouthwashes (e.g., “magic or miracle” solution with viscous lidocaine 2%, diphenhydramine and/or aluminum hydroxide or magnesium hydroxide) [105], topical analgesics (e.g., benzocaine, butyl aminobenzoate, menthol, phenol or tetracaine hydrochloride), coating agents, topical steroidal anti-inflammatories (e.g., clobetasol gel 0.05% or dexamethasone 0.1 mg/mL in patients with multiple or not easily reachable lesions), non-steroidal anti-inflammatories (e.g., amlexanon 5% oral paste), or systemic analgesics (1, 98).The SWISH trial showed that the administration of a topical dexamethasone solution was effective in the treatment of mIAOM in breast cancer patients receiving everolimus in combination with exemestane [52]. After 8 weeks, the incidence of grade ≥ 2 stomatitis was only 2%, compared with the historical data of 33% of the BOLERO-2 study (*p* = 0.0001), with no grade 3 toxicity [52]. Moreover, a temporary interruption of treatment associated with the administration of a topical dexamethasone solution, followed by topical miconazole gel, have proved effective in the treatment of mIAOM in breast cancer patients receiving everolimus in combination with exemestane (Figure 2) [106].

If severe pain occurs, amlexanox 5% oral paste and an early use of fast acting opioids could be considered [51]. A novel and potentially useful approach in this case could be the use of low-level laser therapy [102,107].

#### 5.3.2. EGFR/HER-1 Inhibitors

Epidermal growth factor receptors (EGFR) play a key role in homeostasis, hence their inhibition causes mucosal toxicity in 15% of treated patients. Among EGFR inhibitors, afatinib causes the higher rate of all-grade mucositis (25–72.1%). Cetuximab and panitumumab have an all-grade OM incidence of 7% and 5%, respectively; only 1% of patients experience ≥ grade 3 toxicity [108,109]. This significantly rises when combined with CT [102].

Clinical presentation: Lesions appear as limited, well-defined superficial ulcers with a modest erythema involving non-keratinized oral mucosa, with a pattern not dissimilar to lesions induced by mTOR inhibitors. Lip erythema, erosions, cracks and angular cheilitis are quite common. Deeper mucosal lesions are not very frequent; they only occur during combination treatment. EGFR-induced mucositis is associated with mucosal hypersensitivity/dysesthesia and dysgeusia, and is sometimes painful. They appear shortly after starting treatment, and rapid onset and gradual disappearance has been reported [102].

Management: Management is based on MASCC guidelines for CT and RT. As recommended by the ESMO guidelines, steroids (topical, intralesional or systemic) are considered the first line of therapy; 0.2% morphine mouth-wash or doxepin rinses may be useful in patients with ≥G3 OM [51]; in the latter case temporary discontinuation of drugs and dosage adjustment should be considered.

#### 5.3.3. Anti-HER2 Agents 

Recent studies reported, during treatment with the antibody drug conjugate ado-trastuzumab emtansine (TDM-1), the development of mucosal and cutaneous telangiectasias similar to Osler–Weber–Rendu syndrome [102,110,111]. Approximately 30% of patients treated with TDM-1 developed epistaxis, GI or gynecological bleeding despite the absence of thrombocytopenia. These events may, at least in part, be justified by mucosal vascular alterations. Hence, an early screening for mucosal telangiectasia should be performed.

Clinical presentation: Lips, palate, tongue and jugal mucosa are the sites most affected. During diascopy they become blanch, with a domed form and expanded vessels [102]. 

#### 5.3.4. VEGF/VEGFR Inhibitors

This class of drugs includes angiogenesis inhibitors and tyrosine kinase inhibitors (TKIs). The incidence of mucositis with these agents is very low. Nevertheless, dysgeusia, xerostomia and aphtha may occur, mainly with TKIs. Geographic tongue has been reported during treatment with bevacizumab or TKIs. Sunitinib and cabozantinib induce all-grade OM (34.21–40.08%) [112]; however, dose adjustment or interruption is rarely needed.

Clinical presentation: Xerostomia, discomfort and taste changes, in absence of morphological changes, are the most common clinical presentation. Symptoms appear in the first two months of treatment and then progressively disappear. Some studies showed a correlation between OM and hand–foot syndrome [113,114].

Management: Rinses with benzydamine HCl have shown efficacy for pain relief [115]. Treatments such as antifungal agents must not be prescribed because they are ineffective [116]. 

#### 5.3.5. BRAF Inhibitors

Mucositis induced by BRAF inhibitors has only been reported recently; the incidence is unknown but it seems an uncommon side effect. Mucosal damage is secondary to hyperkeratotic stimulus of keratinized and non-keratinized mucosa and often occurs within the first weeks of treatment [102].

Clinical presentation: All lesions exhibit similar features—asymptomatic, hyperkeratotic, multifocal and with rapid onset—and sometimes have a verrucous or papillomatous appearance at the level of the linea alba, tongue and labial mucosa [102].

Management: By blocking the downstream MAPK pathway, MEK inhibitors significantly restrict the development of secondary hyperkeratotic lesions. There is, otherwise, no specific management for these lesions, apart from a topical one.

#### 5.3.6. CDK4/6 Inhibitors

CDK4/6 inhibitors act by preventing the release of the Retinoblastoma (Rb) protein, thus arresting the cell cycle in a quiescent phase. Gastrointestinal epithelium is the most affected tissue. Preclinical studies showed that the binding of cyclin D3 to CDK4/6 was essential for the proliferation of GI epithelial cells. The mucosal damage was either a direct effect of these drugs or a functional defect appearing in some enzymes, or as disturbances in cellular response to injury and interplay with Wnt/β -catenin, MAPK and NF-κB physiologically expressed in GI cells [117,118].

In patient receiving CDK4/6 inhibitors the incidence of all-grade OM was low, 1% ≥ G3 OM [119]. No high-grade stomatitis required dose reductions or treatment discontinuation.

Studies evaluating ribociclib and palbociclib showed a low occurrence of diarrhea. Conversely, in MONARCH trials, abemaciclib induced early-onset GIM in approximately 70–90% of patients, usually in the first treatment cycles [120].

Clinical presentation: Stomatitis is characterized by aphthous ulcers, usually of mild–moderate severity [121]. The median duration of GIM for abemaciclib was 7.5 days (G2) and 4–5 days (G3) [119]. A significant decrease in severe diarrhea after cycle 4 and 5 has been observed [119].

**Figure 3:**
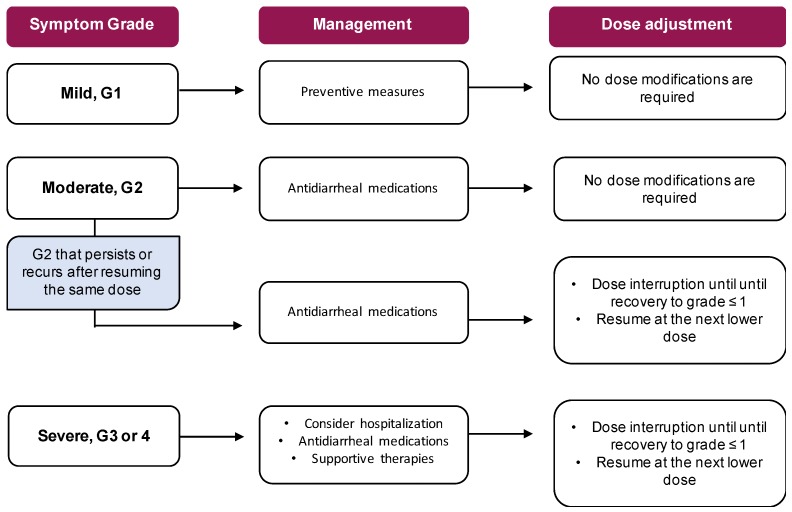
Dose modifications and management of CDK4/6 inhibitors.

Management: Clinical experience with these drugs is still limited and data on their proper management are not yet available. Blood tests may identify alterations in electrolyte levels. [122]. Nausea and vomiting could be treated with the usual antiemetics (i.e., metoclopramide, serotonin 5-HT3 antagonists) if necessary [122]. However, clinicians should be aware of potential interactions between ribociclib and co-medications, due to the risk of QTc prolongation. Upfront management of diarrhea includes hydration and dietary modification. If needed, the treatment could be extended to antidiarrheal agents (loperamide, diphenoxylate/atropine, octreotide, etc.) (Figure 3).

### 5.4. Immunotherapy

Immunotherapy-related adverse events (irAEs) are caused by the stimulation and recruitment of the immune system. IrAEs include autoimmune enteropathy and/or autoimmune colitis and inflammatory bowel disease-like colitis. Most clinical trials have reported GI tract toxicities as the most common serious irAE, often leading to discontinuation of treatment. Regimens containing CTLA-4 agents are more likely to cause GI toxicities (27–54%), potentially determining drug discontinuation [123,124].

Very few data are available on the GI irAES caused by anti-programmed death 1 (PD1) and anti- anti-programmed death ligand 1 (PDL1). Usually, grade 3 and 4 occur in 1–2% of cases [125,126]. However, a spectrum of associated adverse oral events has recently emerged.

Use of both PD-L1 and PD-1 inhibitors has been associated with nonspecific stomatitis or oral mucosal inflammation in sporadic cases; no grade ≥ 3 side effects have been observed. Recently, oral lesions more characteristic of PD-1 or PD-L1 inhibitors have been reported.

Histopathological analysis of the mucosal lesions associated with anti-PD1 and anti-PDL1 therapy revealed an abundant histiocytic and T cell infiltration.

Clinical Presentation: Xerostomia, dysgeusia and lichenoid reactions, possibly associated with pain, generally occur several months after initiation of treatment [102,127]. The lesions may appear as whitish papules in reticular or linear streaks.

GI mucosa could be congested, erythematous and granular in endoscopy, sometimes accompanied by nausea, vomiting, fatigue, and weight loss [102].

The onset of diarrhea varies from 1 to 19 weeks, with a median interval of 3 months after starting treatment, more specifically from 1 to 10 infusions for anti-CTLA4 and approximately 3 months for anti-PD1 and anti-PDL1 [128,129]. Diarrhea is the most common symptom; it can be severe and/or hemorrhagic, with abdominal pain, constipation, anorexia and dehydration [130,131]. Endoscopically, colic mucosa could be ulcerated and friable, either diffusely or unevenly, mainly during anti-CTLA4 treatment [102]. Conversely, anti-PD1/anti-PDL1 endoscopic findings comprise either normal mucosa or inflammatory changes ranging from mild erythema to severe inflammation. Common laboratory findings are anemia, increased serum C-reactive protein and low serum albumin level [132].

**Figure 4:**
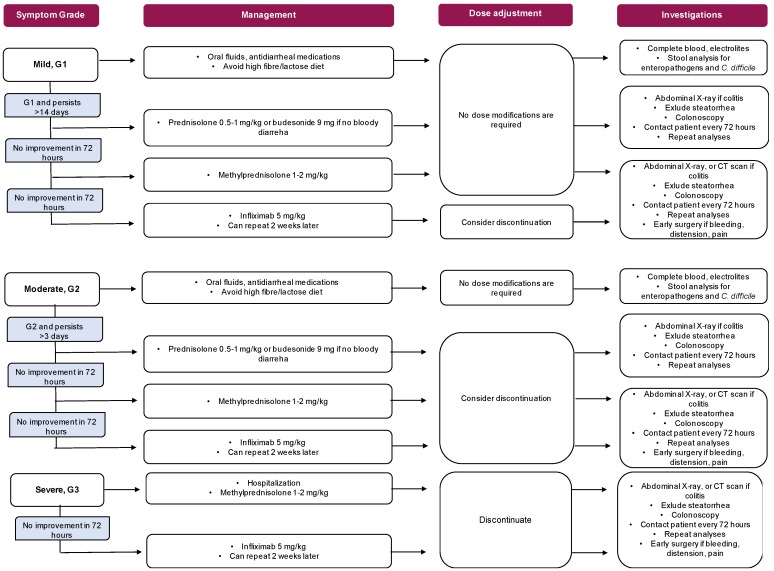
Dose modifications and management of immunotherapy.

Management: Two randomized trials exploring prevention of anti-CTLA4 induced enterocolitis failed to demonstrate any benefit from oral budesonide [133,134]. Patients with diarrhea should undergo a comprehensive clinical and laboratory assessment, including complete blood count, serum electrolytes, stool analyses for enteropathogens and Clostridium difficile. If non-severe diarrhea occurs, an antidiarrheal, fluids and electrolyte supplementation should be administered [123]. Conversely, in the case of severe diarrhea, immunotherapy should be discontinued and systemic corticosteroids administered (1–2 mg/kg per day, intravenous i.v.). After 3–5 days of i.v. corticosteroids, the treatment should be switched to infliximab (5mg/kg in a single dose). A second dose of infliximab after 2 weeks may be needed in non-responsive patients (Figure 4) [123,124,128,129].

## 6. A Comprehensive Management

### 6.1. Pain Management

Management of oral pain is essential to improving the quality of life of patients experiencing CT-induced mucositis. Considering the lack of studies in this setting, clinical guidelines for the management of pain should be considered, adopting individualized approaches. Opioids, non-opioids and adjuvant medications might be considered, preferably with transdermal or intravenous administration.

Morphine seems to require relatively lower doses and might be better tolerated than other opioids [50]. Notably, 0.2% morphine mouthwash may be effective in patients undergoing CT-RT for the treatment of pain due to OM [50].

Topical therapy with doxepin rinse, a tricyclic antidepressant with anesthetic and analgesic potential, has been evaluated in patients receiving RT for HN malignancies, and HSCT and its use has been observed to significantly reduce the intensity of pain [135]. A randomized, double-blind, placebo-controlled trial testing the efficacy of doxepin in terms of the reduction of RT-induced pain revealed that doxepin determined a greater reduction in mean mouth and throat pain compared to the placebo (*p* < 0.001) [136]. Doxepin hydrochloride mouthwash may therefore be effective to treat pain related to OM in all types of cancer and treatment modalities [50,65].

Fentanyl may be effective to treat pain due to OM in patients treated with CT, with or without RT [50]. A single-center study showed that transdermal fentanyl reduces the mean pain scores when administered at 25 μg/h during CT-RT [137].

Lastly, different topical preparations have been evaluated. Most frequently, these include lidocaine, benzocaine, milk of magnesia, kaolin, pectin, chlorhexidine, and diphenhydramine. However, there is no significant evidence to support their effectiveness or tolerability and the absorption of amine anesthetics (e.g., lidocaine) through damaged mucosa may in fact exacerbate the toxicity [65].

### 6.2. Treatment of Infections

When mucosal damage is established, oral ulcers may easily become a portal for bacterial and fungal invasion, potentially leading to systemic infections in neutropenic patients. The use of chlorexidine has been studied as a prophylactic strategy inferior to cryotherapy in reducing the frequency and duration of severe mucositis, while no data are available for the “on-treatment” settings [58].

Other antimicrobial agents, including miconazole and fluconazole, have recently been considered for the treatment of mucositis [138,139]. Nevertheless, there is currently no conclusive evidence to support the use of miconazole or fluconazole in treating CT-induced mucositis and future randomized, double-blind, placebo-controlled clinical trials are needed to evaluate their potential effectiveness in the treatment of this toxicity.

### 6.3. Nutritional Assessment and Implementation 

Nutritional screening and regular follow-up should be offered to patients during anti-cancer treatment [140]. Available clinically validated tools include: the malnutrition universal screening tool (MUST), the malnutrition screening tool (MST), the patient-generated subjective global assessment (PG-SGA), nutritional risk screening (NRS 2002), the mini nutritional assessment (MNA) and the NUTRISCORE tool.

In the event of OM or GIM, early recognition and nutritional intervention are required. Patients with OM usually benefit from a soft diet, irritating food must be avoided. [140]. Oral nutritional supplements (ONS) should be considered when dietary measures are not sufficient [141].

In the event of severe undernourishment, supplemental artificial nutrition may be offered. This should be considered particularly when food intake is predicted to be less than 60% of the estimated expenditure for more than 10 days [141].

## 7. Conclusions

Mucositis is the most common debilitating side effect related to chemotherapy (CT), radiation therapy (RT), targeted agents, and immunotherapy. An adequate classification of lesions is crucial in choosing the most suitable management and, eventually, the appropriate dose modifications of cancer therapy. Understanding the pathophysiology and management of mucosal injury as a secondary effect of anti-cancer treatment is an important area of clinical research; henceforth, prophylaxis, early diagnosis, and adequate management of complications are essential to increase therapeutic success and, thus, improve survival outcomes of cancer patients.

## Figures and Tables

**Table 1:** Prophylaxis strategies for oral and gastrointestinal mucositis.

Intervention	Aim	Treatment Setting	Ref.
Prevention of OM			
Basic oral care protocol	Prevention of OM	All cancer treatments	[50,51]
Oral cryotherapy	Prevention of OM	Bolus 5-FU chemotherapy HD melphalan ± TB-RT for HSCT	[50,51] [50,51]
Benzydamine mouthwash	Prevention of OM	RT for HN cancer patients	[50,51]
Photobiomodulation (PBM)	Prevention of OM	HDCT ± TB-RT for HSCT RT for HN cancer patients	[50,51] [50,51]
Palifermin	Prevention of OM	HDCT and TB-RT for HSCT	[50,51]
Zinc supplements	Prevention of OM	RT or CT	[50,51]
Prevention of mIAS			
Dexamethasone-containing mouthwashes	Prevention of mIAS	BC patients treated with everolimus	[52]
Prevention of GIM			
Amifostine	Prevention of xerostomia	Post-operative RT for HN cancer patients	[53]
	Prevention of proctitis	RT for pelvic malignancy	[50,51]
	Prevention of esophagitis	Concomitant CT-RT in NSCLC	[50,51]
Sulfasalazine	Prevention of enteropathy	Pelvis RT	[50,51]
Probiotics	Prevention of diarrhea	CT and/or RT for pelvic malignancy	[50,51]

Legend: OM—oral mucositis; mIAS—mTOR inhibitor-associated stomatitis; GIM—gastrointestinal mucositis; FU—florouracil; HD—high-dose; TB—total body; RT—radio therapy; HSCT—hematopoietic stem cell transplantation; HN—head and neck; BC—breast cancer; HDCT—high-dose chemotherapy; NSCLC—non-small cell lung cancer; CT—chemotherapy.
